# Factors Affecting the Length of Hospital Stay in Hypercapnic Respiratory Failure

**DOI:** 10.3390/diagnostics15010014

**Published:** 2024-12-25

**Authors:** Maşide Ari, Tarkan Ozdemir, Murat Yildiz, Deniz Celik, Eren Usul, Emrah Ari, Ömer Faruk Tüten

**Affiliations:** 1 Clinic of Lung Diseases, Ankara Atatürk Sanatory Education and Research Hospital, 06290 Ankara, Türkiye; drmuratyildiz85@gmail.com; 2 Clinic of Lung Diseases, Konya Farabi Hospital, 42090 Konya, Türkiye; tarkanozdemir78@gmail.com; 3 Clinic of Lung Diseases, Alanya Alaaddin Keykubat University Education and Research Hospital, 07400 Antalya, Türkiye; drdenizcelik@hotmail.com; 4 Department of Emergency Medicine, Etlik City Hospital, 06170 Ankara, Türkiye; usuleren7@hotmail.com; 5 Department of Emergency Medicine, Mamak Public Hospital, 06620 Ankara, Türkiye; dremrahari25@gmail.com; 6 Clinic of Lung Diseases, Ankara University Health Practise and Research Hospitals, 06050 Ankara, Türkiye; omertuten@gmail.com

**Keywords:** albumin, CCI, COPD, HRF, length of stay, ICU

## Abstract

**Background/Objectives:** Hypercapnic respiratory failure (HRF) is a primary cause of admittance to the intensive care unit (ICU). This study aimed to investigate the factors that affect the length of hospital stay in HRF patients. **Methods:** This study was designed as a retrospective, cross-sectional analysis of patients who were admitted to the ICU because of HRF between 2022 and 2024. The demographic and clinical characteristics of the patients and laboratory results were recorded. The Charlson Comorbidity Index (CCI) was calculated. The relationship between these parameters and the length of hospital stay was assessed. **Results:** A total of 138 patients were included in the study. The average length of hospital stay was 11.45 days, and 37% of the patients were included in the long-term hospitalization group. The degree of hypercapnia was not associated with the length of hospital stay. It was determined that the patients’ albumin levels and CCI were significant determinants of the length of hospital stay. The combined assessment of these two parameters was found to be superior compared to their separate evaluations. **Conclusions:** In our study, hypoalbuminemia and a higher CCI were identified as predictors of a prolonged ICU stay in HRF patients. Albumin levels of <3.25 g/dL and CCI scores of ≥5 were linked to longer stays, with this combined evaluation offering greater predictive value. These factors can guide patient management.

## 1. Introduction

The intensive care unit (ICU) is a crucial aspect of the healthcare system, providing specialized and costly medical care for patients in a critical condition. Because of the limited bed capacity of ICUs, it is essential to use these resources effectively. It is important to ensure that patients are discharged from the ICU at the correct time, especially when the occupancy rates are high. However, patients in the ICU tend to have prolonged hospital stays due to the severity of their illnesses. Understanding the factors that influence patients’ duration of stay in the ICU can help to optimize patient management and resource allocation.

Hypercapnic respiratory failure (HRF) is a type of respiratory failure that is characterized by inadequate ventilation, leading to levels of pCO2 in arterial blood exceeding 45 mmHg. It is one of the leading causes of admittance to the intensive care unit (ICU). Many studies have examined the success of NIMV (non-invasive mechanical ventilation) in patients with HRF and its effect on the prognosis of the disease [1]. However, the number of studies examining the factors that affect these patients’ length of stay in the ICU is not sufficient. Studies have shown that a prolonged hospital stay in these patients, regardless of the cause, increases the risk of mortality [2].

The Charlson Comorbidity Index (CCI) is a weighted scoring system that includes 19 different medical categories, each weighted according to its potential impact on mortality [3]. It is frequently used to determine the risk of mortality and perform a prognostic evaluation on patients. Hasegawa et al. emphasized the prognostic importance of the CCI in their study evaluating surgical success in pancreatic cancer [4]. In a study conducted on patients with heart failure, it was found that a high CCI was associated with readmission [5]. HRF is the result of many conditions, and comorbidity is common among these patients.

HRF is one of the most significant causes of admittance to the intensive care unit. Identifying patients who are likely to have a prolonged stay in the ICU would enable us to reduce nosocomial infection, cognitive dysfunction, and other morbidities. In addition, it is important to understand the factors that affect patients’ length of stay in the ICU in advance because this would enable the implementation of personalized interventions for those that can be treated. Therefore, the effects of the CCI and other parameters on the length of hospital stay were examined in our study.

## 2. Materials and Methods

This paper presents a retrospective study of patients with hypercapnic respiratory failure who were admitted to the Pulmonary Diseases Intensive Care Unit of the Ankara Atatürk Sanatorium Training and Research Hospital between January 2022 and May 2024. It was conducted following the ethical principles outlined in the Declaration of Helsinki, after being approved by the Ankara Atatürk Sanatorium Training and Research Hospital’s Clinical Research Ethics Committee, with decision number 111, dated 17 July 2024.

Approximately 1000 patients are admitted to the Pulmonary Diseases Intensive Care Unit of Ankara Atatürk Sanatorium annually. Most patients have underlying lung diseases, with approximately 35% experiencing hypercapnic respiratory failure.

The patients in this study received medical treatment according to established guidelines. BiPAP was applied using an oronasal or full-face mask in the treatment of hypercapnic respiratory failure. Respiratory support was provided using a non-invasive mechanical ventilator in BiPAP-ST and AVAPS modes, with the tidal volumes calculated based on the patients’ estimated bodyweight. The patients were closely monitored, receiving oxygen support to ensure that saturation was maintained at between 90 and 92%. When assessing patients’ responses to treatment, their arterial blood gas (ABG) and vitals, such as their respiratory rate, blood pressure, and heart rate, were measured. The ABGs of the patients were recorded at the time of admission or at the first admission to the ICU and at discharge. At follow-up, patients with a confirmed need for long-term NIMV, based on their arterial blood gas results, were provided with BiPAP-ST devices by the Ministry of Health. Once the patients had adapted to the device in the ICU, they were discharged home with the device.

The patients’ length of hospital stay in the ICU was recorded, and the average duration was calculated. Patients whose length of hospital stay was below the median value were included in the short-term hospitalization group, and patients whose length of hospital stay was at or above the median value were included in the long-term hospitalization group.

The demographic characteristics of the patients, including their age, sex, comorbid conditions, and medications, were recorded. The Charlson Comorbidity Index was utilized. Subsequently, blood tests and all the treatments administered within the first 24 h of ICU admission were reviewed using the hospital’s automation system and the patients’ physical files. The relationship between these parameters and the patients’ length of hospital stay was analyzed.

### 2.1. Patient Selection

**Figure 1:**
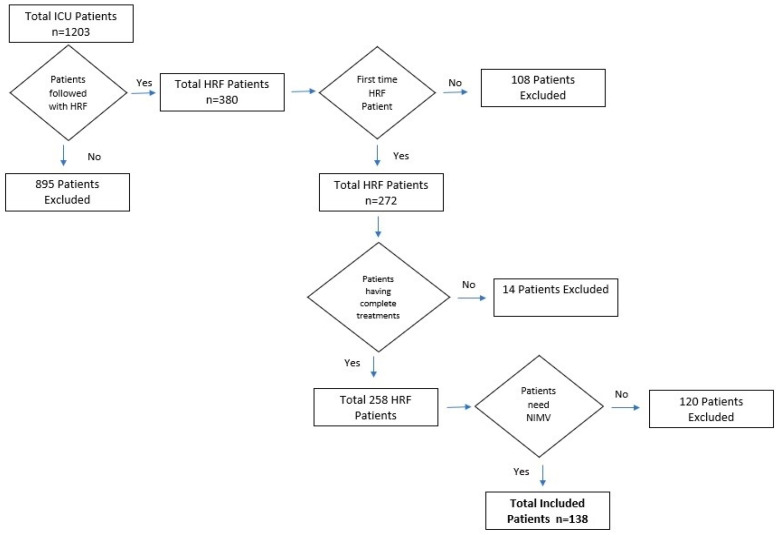
Flowchart of patients included in and excluded from this study.

This study included patients who experienced hypercapnic respiratory failure (pCO_2_ > 45; HRF may or may not be accompanied by decompensation) for the first time and were subsequently monitored in the Chest Diseases ICU during the specified dates. Patients with a prior history of hypercapnic respiratory failure, those using home non-invasive mechanical ventilation (NIMV), patients who no longer required NIMV during follow-up or at discharge, those with a hospital stay of less than one day, patients who died during follow-up, and those with incomplete data were excluded from the study. Figure 1 shows a flowchart detailing the patients included in and excluded from this study.

### 2.2. Statistical Analysis

The statistical analysis of the data obtained was performed using IBM SPSS Statistics (version 27.0, IBM Inc., Chicago, IL, USA). The normality of the variable distribution was assessed using visual (histograms and probability plots) and analytical methods (Shapiro–Wilk tests). Descriptive statistics are reported as means and standard deviations for normally distributed numerical data, medians and ranges (minima–maxima) for non-normally distributed data, and counts and percentages for nominal data. For non-normally distributed data, the Mann–Whitney U test was used between two groups. For the factors affecting patients’ length of hospital stay, the area under the curve (AUC) was evaluated in ROC analysis, with the data expressed at a 95% confidence interval. In multivariate analysis, the independent variables used to predict the discharge time were examined using logistic regression analysis; this utilized possible factors that had been identified in previous analyses. The Hosmer–Lemeshow test was used to assess the model’s fit. In the statistical analyses, *p*-values below 0.05 were considered as statistically significant.

## 3. Results

A total of 1203 patient files in the ICU were reviewed. Hypercapnic respiratory failure was detected for the first time in 258 patients whose data were fully accessible. Subsequently, 120 patients who no longer required NIMV during follow-up were excluded, resulting in a study population of 138 patients. The demographic characteristics are presented in Table 1.

The average length of hospital stay in the intensive care unit was 11.45 days. Patients who were hospitalized for 11 days or less were included in the short-term hospitalization group. In total, 37% of the patients were in the long-term hospitalization group. The demographic characteristics of the patients according to the length of their hospital stay are given in Table 1.

The results of the laboratory tests performed upon admission to the intensive care unit and their relationship with the patients’ length of hospital stay are presented in Table 2. It was found that the patients’ level of urea was associated with the length of their hospital stay (*p* = 0.023). Additionally, hypoalbuminemia was related to an extended hospital stay (*p* = 0.001) (Table 2).

The results of the tests performed to assess the patients’ arterial blood gas upon admission and discharge are presented in Table 3. The degree of hypercapnia was not associated with an extended hospital stay.

The independent variables used to predict the patients’ length of hospital stay were analyzed using multivariable logistic regression. It was found that albumin and the Charlson Comorbidity Index (CCI) were independent predictors of long-term hospitalization (*p* = 0.001, *p* = 0.044) (Table 4).

**Figure 2:**
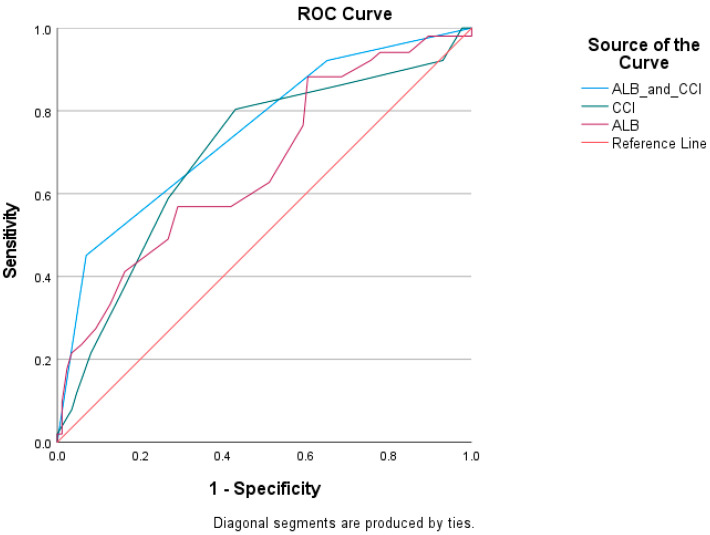
ROC curve used to predict long-term hospitalization.

The albumin levels were found to be 3.4 in the short-term hospitalization group and 3.1 in the long-term hospitalization group, with a significant difference between the groups (*p* = 0.001). The cutoff value for albumin was determined to be 3.25, with a sensitivity of 70.9% and a specificity of 56.9. It was determined that albumin is a determinant associated with the prolongation of hospitalization (AUC: 0.665, 95% CI: 0.571–0.760, *p* < 0.001) (Table 5) (Figure 2).

The Charlson Comorbidity Index (CCI) was found to be 4.60 in the short-term hospitalization group and 5.98 in the long-term hospitalization group, with a significant difference between the groups (*p* < 0.001). The cutoff value for the CCI was determined to be 5, with a sensitivity of 80.4 and a specificity of 57.5%. It was determined that the CCI is a determinant associated with the prolongation of hospitalization (AUC: 0.697, 95% CI: 0.604–0.791, *p* < 0.001) (Table 5) (Figure 2).

When albumin and CCI were evaluated together at a cutoff value of ≥1, a sensitivity of 92.2% and a specificity of 34.9% were found to prolong the hospitalization of patients (AUC: 0.750, 95% CI: 0.664–0.835, *p* < 0.001). The combined assessment of both parameters proved to be superior in determining the patients’ length of hospital stay compared to their separate evaluations (Table 5) (Figure 2).

## 4. Discussion

Intensive care units (ICUs) are critical and high-cost components of modern healthcare systems, providing high-level medical care in life-threatening situations [6]. Because of their limited capacity, ICUs must be used effectively. Therefore, patients’ length of stay in the ICU is a key indicator that can be used to monitor and evaluate the performance of hospitals [7]. Acute respiratory failure due to the exacerbation of COPD is one the primary causes of admittance to the ICU [8]. These exacerbations account for approximately 2% of all ICU admissions [9]. The average length of hospital stay for patients with COPD is reported to range from 5 to 12 days, with this increasing in cases requiring non-invasive mechanical ventilation (NIMV) [10]. A study examining patients’ length of stay in the ICU because of respiratory failure found that the average duration was 9 ± 7 days [11]; however, this study did not consider the patients’ prior history of respiratory failure, existing NIMV support at home, or ongoing NIMV needs. Our study, which is the first to focus on patients with a history of respiratory failure, excluded those who no longer required NIMV. The average length of hospital stay in our study was 11.45 days, suggesting that this duration is within the ideal and expected range, especially given that the need for NIMV extends the length of ICU hospitalization for these patients.

Patients’ length of hospitalization in the ICU has emerged as a fundamental metric for ensuring the efficient use of healthcare resources and reducing the financial burden on healthcare systems [12]. Prolonged hospitalization is associated not only with increased mortality but also with higher risks of hospital-acquired infections and morbidities. Reducing this duration can enhance the quality of treatment, improve bed management, and lower healthcare costs. Despite numerous studies on the success of treatment in respiratory failure, few have assessed patients’ length of hospital stay. In our study, it was observed that patients’ comorbid conditions and hypoalbuminemia prolonged their hospitalization. These results may guide treatments, promote the efficient use of resources, improve patient outcomes, and shorten the length of ICU hospitalization.

Elderly patients have become a significant population in intensive care units because of factors such as frailty, comorbidities, and polypharmacy. Many studies have indicated that a substantial proportion of ICU admissions comprise individuals aged 60 and above [13]. In our study, the average age of the patients was 69.

It is known that managing elderly patients is challenging because of the complexity of their comorbid conditions; thus, a longer hospital stay is often required in order to ensure recovery [14]. A study by Vallet and colleagues indicated that patients’ length of hospital stay is longer in the elderly population [15]. Our study also demonstrated that patients with a longer hospital stay had a higher average age. This condition is thought to be associated with a higher burden of comorbidities.

The effects of gender on diseases and prognoses are a topic of increasing importance [16]. In most patient groups, men are more likely to experience critical illness requiring ICU admission [13]. In a study examining the effects of gender on the need for intensive care and patients’ length of hospitalization, it was reported that most of the patients were male; however, there was no significant difference in terms of hospital stay [17]. In our study, 63.8% of the patients were male; however, no statistically significant difference was observed in the length of hospital stay between the two genders.

Hypercapnic respiratory failure (HRF) is a type of respiratory failure in which pCO2 levels in arterial blood gas are above 45 mmHg, particularly in cases where ventilation is not sufficient. Hypercapnic respiratory failure is caused by many factors. The relationship between the degree of HRF and the success of non-invasive mechanical ventilation (NIMV) has been studied frequently [18]; however, its impact on patients’ length of hospital stay has not been evaluated. In our study, the degree of hypercapnia and pH were not found to be associated with the patients’ length of hospital stay. This was associated with the similarity of the pCO_2_ and pH levels in these patients admitted to the intensive care unit.

The indicators commonly used to assess kidney function include the glomerular filtration rate and serum creatinine and urea levels [19]. Urea is a known product of protein catabolism. Patients requiring intensive care are recognized to be in a catabolic state [20]. In critically ill patients with a high level of protein catabolism, increases in urea levels are more pronounced than those in serum creatinine levels, suggesting that urea has a higher predictive value. Elevated urea levels present in aspiration pneumonia have also been associated with poor outcomes [21]. A study by Du and colleagues on COPD exacerbations found that higher urea levels were linked to prolonged hospitalization [22]. Our study also demonstrated that patients with prolonged hospitalization had higher urea levels.

Serum albumin is considered to be a marker of disease severity [23]. A study by Sai and colleagues noted that there is a relationship between hypoalbuminemia and patients’ length of hospital stay [24]. In our study, the cutoff value for albumin was determined to be 3.25 g/dL, showing high sensitivity (70.9%) and moderate specificity (56.9%) in predicting prolonged hospitalization. Low albumin levels were demonstrated to be an independent predictor of extended hospitalization, consistent with existing literature that highlights its role as an indicator of poor nutritional status and increased morbidity. This emphasized the importance of closely monitoring albumin levels in patients at risk and may also guide personalized treatments.

The concept of comorbidity was first defined by Alvan R. Feinstein [25]. Later, the Charlson Comorbidity Index (CCI), defined by Charlson, became the gold standard for assessing comorbid conditions in clinical research [3]. The CCI is a validated index that predicts the risk of mortality associated with comorbid diseases, and studies have indicated its association with patients’ length of stay in the ICU [26,27]. The CCI has been studied mostly in the evaluation of NIMV success in HRF [28]. However, its impact on patients’ length of hospital stay has not been extensively studied. Our study found that when using a cutoff value of 5, the CCI serves as a high-sensitivity independent predictor (80.4%) for prolonged hospitalization. Higher CCI scores were linked to greater comorbidity and prolonged hospitalization, supporting the idea that comorbid conditions complicate patient management and recovery, thereby extending the duration of care. Comorbid conditions are an important parameter in disease management, as they can change not only the prognosis of the disease but also the treatment results.

When evaluating the patients’ length of hospital stay, the combination of the CCI and albumin increased our ability to predict prolonged hospitalization to 92.2%, despite a drop in the specificity to 34.9%. In addition, because of the high negative predictive value, the use of both parameters together increases the accuracy for predicting long-term hospitalization. The results of this study indicate that a high CCI score and hypoalbuminemia significantly prolong the duration of hospitalization. This suggests the potential for targeted treatments and the more effective use of resources.

The aging population, increased life expectancy, and rising number of patients requiring prolonged hospitalization when admitted to the ICU pose an escalating challenge for healthcare systems. Therefore, identifying the factors that affect patients’ length of hospital stay will be crucial for guiding personalized treatments. Our study demonstrated that albumin and the CCI are significant predictors of patients’ length of hospital stay, both of which are commonly used parameters in intensive care. However, their impacts on patients’ length of hospital stay in cases of hypercapnic respiratory failure (HRF) have not yet been studied. This research is the first to illustrate the effects of the CCI and albumin on the length of hospital stay in patients with HRF, and we believe it will serve as a guide for future studies.

Our study has some limitations. Discharge times may have been prolonged for certain patients because of social support and other factors, but these may have been overlooked because of the retrospective nature of this study. Additionally, because this research was conducted at a single center, it consists of patients with similar sociocultural backgrounds. Multicenter studies would further enhance the value of our findings.

## 5. Conclusions

This study highlights the critical factors affecting the length of hospital stay in patients with HRF monitored in the ICU. Our findings reveal that hypoalbuminemia and higher CCI scores are independent predictors of prolonged hospitalization. This underscores the importance of assessing both nutritional status and comorbid conditions upon ICU admission. The results of our study have the potential to guide personalized treatment plans, optimize resource allocation, and improve overall patient outcomes. Furthermore, considering the increasing ICU admissions associated with an aging population, these findings may serve as a guide for future strategies aimed at enhancing healthcare efficiency.

## Figures and Tables

**Table 1:** Demographic characteristics of the patients according to the length of their hospital stay.

Demographic Characteristic	All Patients (*n* = 138) (100%) (Mean ± SD)	Short-Term Hospitalization Group (*n* = 87) (63%) (Mean ± SD)	Long-Term Hospitalization Group (*n* = 51) (37%) (Mean ± SD)	*p*-Value *
Age, years (Mean ± SD)	69 ± 10	67 ± 9	71 ± 11	**0.024**
Length of Hospital Stay	11.45 ± 4.96 (days)	8.37 ± 1.77 (days)	16.71 ± 4.18 (days)	
Gender				
Female	50 (36.2%)	28 (43.1%)	22 (32.2%)	0.198
Male	88 (63.8%)	59 (56.9%)	29 (67.8%)	
Comorbidity				
Hypertension	71 (51.4%)	44 (50.6%)	27 (52.9%)	0.789
Coronary Artery Disease	27 (19.6%)	13 (14.9%)	14 (27.5%)	0.075
Diabetes Mellitus	37 (26.8%)	19 (21.8%)	18 (35.3%)	0.086
Atrial Fibrillation	20 (14.5%)	9 (10.3%)	11 (21.6%)	0.072
Chronic Kidney Disease	24 (17.4%)	11 (12.6%)	13 (25.5%)	0.056
Bronchiectasis	9 (6.5%)	5 (5.7%)	4 (7.8%)	0.632
Lung Cancer	9 (6.5%)	5 (5.7%)	4 (7.8%)	0.632
Emphysema	40 (28.9%)	27 (33.3%)	13 (26.5%)	0.417
CCI **	5.11 ± 2	4.60 ± 1.90	5.98 ± 2.20	<0.001
History of Long-term Oxygen Use	98 (71%)	66 (75.9%)	32 (62.7%)	0.102

* Mann–Whitney U analysis was applied. ** CCI: Charlson Comorbidity Index.

**Table 2:** Evaluation of laboratory tests performed upon admission to the ICU.

Laboratory Finding	All Patients (*n* = 138) Median (Interquartile Range (25–75))	Short-Term Hospitalization Group (*n* = 87) Median (Interquartile Range (25–75))	Long-Term Hospitalization Group (*n* = 51) Median (Interquartile Range (25–75))	*p* Value *
Urea (mg/dL)	51 (34–79)	44 (32–64)	63 (38–89)	0.023
Creatinine (mg/dL)	1.03 (0.66–1.29)	1.01 (0.78–1.26)	1.07 (0.79–1.36)	0.537
Albumin (g/dL)	3.4 (2.6–3.7)	3.4 (3.1–3.8)	3.2 (2.8–3.5)	0.001

* Mann–Whitney U analysis was applied.

**Table 3:** Arterial blood gas results at admission and discharge.

Blood Gas	All Patients (*n* = 138) Median (Interquartile Range (25–75))	Short-Term Hospitalization Group (*n* = 91) Median (Interquartile Range (25–75))	Long-Term Hospitalization Group (*n* = 47) Median (Interquartile Range (25–75))	*p*-Value *
Admission Blood Gas				
pH	7.30 (7.24–7.36)	7.30 (7.24–7.36)	7.31 (7.24–7.36)	0.788
pCO_2_	79 (69–93)	80 (72–93)	77 (66–92)	0.139
HCO_3_	38 (34–45)	39 (35–45)	37 (31–46)	0.247
Discharge Blood Gas				
pH	7.46 (7.43–7.50)	7.46 (7.43–7.50)	7.45 (7.42–7.49)	0.454
pCO_2_	52 (47–57)	53 (47–56)	52 (47–57)	0.822
HCO_3_	37 (33–40)	37 (33–40)	36 (33–40)	0.901

* Mann–Whitney U analysis was applied.

**Table 4:** Results of logistic regression analysis performed to identify the factors associated with long-term hospitalization.

Variable	*p*-Value	Odds Ratio (95% CI)
Age	0.418	0.979 (0.929–1.031)
Urea	0.870	1.001 (0.988–1.015)
CCI *	0.044	1.366 (1.063–1.756)
Albumin	0.001	0.845 (0.764–0.934)

* CCI: Charlson Comorbidity Index.

**Table 5:** Results of ROC analysis performed to predict long-term hospitalization.

	AUC	95% Confidence Interval	Sensitivity	Specificity	PPV	NPV	LR+	LR−	*p*-Value
Albumin	0.665	0.571–0.760	70.9%	56.9%	73.5%	53.7%	1.64	0.51	<0.001
CCI *	0.697	0.604–0.791	80.4%	57.5%	52.6%	83.3%	1.89	0.34	<0.001
Albumin and CCI	0.750	0.664–0.835	92.2%	34.9%	45.6%	88.2%	1.42	0.22	<0.001

* CCI: Charlson Comorbidity Index.

## Data Availability

The original contributions presented in this study are included in the article. Further inquiries can be directed to the corresponding author.
